# Genetic Insights into the Molecular Pathophysiology of Depression in Parkinson’s Disease

**DOI:** 10.3390/medicina59061138

**Published:** 2023-06-13

**Authors:** Efthalia Angelopoulou, Anastasia Bougea, Yam Nath Paudel, Vasiliki Epameinondas Georgakopoulou, Sokratis G. Papageorgiou, Christina Piperi

**Affiliations:** 1 Department of Neurology, Eginition University Hospital, National and Kapodistrian University of Athens, 11528 Athens, Greece; angelthal@med.uoa.gr (E.A.); annita139@yahoo.gr (A.B.); sokpapa@med.uoa.gr (S.G.P.); 2 Department of Biological Chemistry, Medical School, National and Kapodistrian University of Athens, 75 M. Asias Street, 11527 Athens, Greece; 3 Neuropharmacology Research Laboratory, Jeffrey Cheah School of Medicine and Health Sciences, Monash University Malaysia, Bandar Sunway, Subang Jaya 46150, Selangor, Malaysia; yam.paudel@monash.edu; 4 Department of Infectious Diseases-COVID-19 Unit, Laiko General Hospital, 11527 Athens, Greece; vaso_georgakopoulou@hotmail.com

**Keywords:** Parkinson’s disease, depression, SLC6A4 polymorphism, BDNF, genetic factors, therapeutic target

## Abstract

*Background and Objectives*: Parkinson’s disease (PD) is a clinically heterogeneous disorder with poorly understood pathological contributing factors. Depression presents one of the most frequent non-motor PD manifestations, and several genetic polymorphisms have been suggested that could affect the depression risk in PD. Therefore, in this review we have collected recent studies addressing the role of genetic factors in the development of depression in PD, aiming to gain insights into its molecular pathobiology and enable the future development of targeted and effective treatment strategies. *Materials and Methods*: we have searched PubMed and Scopus databases for peer-reviewed research articles published in English (pre-clinical and clinical studies as well as relevant reviews and meta-analyses) investigating the genetic architecture and pathophysiology of PD depression. *Results*: in particular, polymorphisms in genes related to the serotoninergic pathway (sodium-dependent serotonin transporter gene, *SLC6A4*, tryptophan hydrolase-2 gene, *TPH2*), dopamine metabolism and neurotransmission (dopamine receptor D3 gene, *DRD3*, aldehyde dehydrogenase 2 gene, *ALDH2)*, neurotrophic factors (brain-derived neurotrophic factor gene, *BDNF*), endocannabinoid system (cannabinoid receptor gene, *CNR1*), circadian rhythm (thyrotroph embryonic factor gene, *TEF*), the sodium-dependent neutral amino acid transporter B(0)AT2 gene, *SLC6A15*), and *PARK16* genetic locus were detected as altering susceptibility to depression among PD patients. However, polymorphisms in the dopamine transporter gene (*SLC6A3*), monoamine oxidase A (*MAOA*) and B (*MAOB*) genes, catechol-O-methyltransferase gene (*COMT*), *CRY1*, and *CRY2* have not been related to PD depression. *Conclusions*: the specific mechanisms underlying the potential role of genetic diversity in PD depression are still under investigation, however, there is evidence that they may involve neurotransmitter imbalance, mitochondrial impairment, oxidative stress, and neuroinflammation, as well as the dysregulation of neurotrophic factors and their downstream signaling pathways.

## 1. Introduction

Parkinson’s disease (PD) affects approximately 1–2% of the population and represents the most common neurodegenerative movement disorder over the age of 65 years [1,2]. The cardinal motor features of PD involve bradykinesia, resting tremor, postural instability, and muscular rigidity, but non-motor manifestations, such as psychiatric symptoms, autonomic dysfunction, hyposmia, cognitive impairment, and sleep disorders, are also usually present [3]. There is no approved disease-modifying therapy for PD, and treatment mainly involves levodopa and dopaminergic agonists [2,3]. Dopaminergic treatment provides only partial symptomatic relief for motor symptoms for a limited period of time, and PD patients usually develop debilitating levodopa-induced motor complications (dyskinesias, fluctuations) after its prolonged use [2].

The pathological hallmarks of PD include the nigrostriatal dopaminergic neuronal loss in substantia nigra pars compacta (SNpc), projecting to the striatal region of the basal ganglia, and the deposition of Lewy bodies and Lewy neurites consisting of alpha-synuclein [3]. The pathophysiology of PD remains elusive, although several molecular mechanisms have been implicated in its pathogenesis, including mitochondrial dysfunction, autophagy dysregulation, impaired apoptotic pathways, excessive oxidative stress, and neuroinflammation, as well as proteasome dysfunction [4,5]. PD etiology is considered multifactorial, since both environmental and genetic risk factors contribute to its development [3]. Mendelian-inherited monogenic familial forms of PD constitute only 5–10% of all cases [6], including mutations in the *LRRK2*, *SNCA*, *Parkin*, *PINK1*, *DJ1, ATP13A2*, *VPS35*, and *GBA1* genes, which are inherited in a dominant or recessive manner [7]. Additionally, genome-wide association studies (GWAS) have revealed at least ninety independent genetic loci that may affect the susceptibility to sporadic PD, overall contributing to an estimated heritability of 22–27% [8]. PD represents a clinically heterogeneous disorder and there is a growing interest in the elucidation of the genetic contribution to this diversity [9,10].

Depression is one of the most common non-motor manifestations, and the most frequent neuropsychiatric comorbidity in PD [11]. The frequency of depression among PD patients varies greatly in various epidemiological studies, ranging from 3% to 90%, with an approximate mean prevalence of 40% [11,12]. Depressive symptoms have been correlated with worse motor function, cognitive decline, care-giver stress, and lower quality of life for PD patients [13], while the response to pharmacological treatments for PD-related depression is variable. Although PD-related depression shares several common features with the depression not related to PD, it is characterized by less guilt, anhedonia, self-destructive behavior, delusional ideation, and suicide [14,15], potentially reflecting a distinct pathophysiological background. The neurobiology of depression in PD remains obscure, and its pathogenesis is considered multifaceted. It has been proposed that depressive symptoms may partially represent a reactive situation to the motor and possibly concurrent cognitive deficits, since some studies have shown that depression is associated with disease stage, as well as the duration and severity of motor impairment [16,17,18]. However, other studies have failed to support this hypothesis [19], finding that the depression frequency in PD is higher compared to other neurological disorders [20] and depressive symptoms may precede the appearance of motor features [3]. Neurotoxin-induced PD animal models might demonstrate depressive behavior, even before the onset of motor deficits [21]. The degeneration of specific brain regions and the functional impairment of neuronal circuits are also considered to play direct roles in the development of PD-related depression, independently of the degree of motor impairment. Therefore, depression has been suggested to be an integral element of the molecular pathophysiology of PD, and the elucidation of its pathogenesis is crucial for its optimal management.

It is still unclear why some patients with PD develop depression during the disease course and an important genetic component to non-PD-related depression has already been recognized.

Herein, we discuss the emerging impact of genetic factors in the development of depression in PD, aiming to gain insights into its molecular pathophysiology and enable the future development of targeted and effective treatment strategies. Although some mechanisms underlying PD depression have been previously discussed [22], there is no recent review focusing on the possible impact of gene polymorphisms in its pathogenesis. Given the unclear and complex pathophysiology of PD depression and the growing evidence on the contribution of genetic factors in its development, discussion of the implicated molecular mechanisms from a genetic point of view would be useful for our deeper understanding of this clinical entity. This might also pave the way for future research. To the best of our knowledge, there is so far no recent review following this novel combinational approach in the case of depression in PD. In this review, after a brief description of the diverse clinical profiles of the rarer genetic forms of PD related to depression that have been previously published [6], we mainly focus on the potential impact of gene polymorphisms on the neurobiology of depression in the sporadic-idiopathic forms of PD. Moreover, we propose future research directions that could aid in the clarification of the role of genetic factors in PD depression and the molecular mechanisms involved, based on recent relevant pre-clinical and clinical evidence.

To this end, we searched PubMed and Scopus databases for peer-reviewed research and review articles investigating and/or discussing the genetic architecture and pathophysiology of PD depression, published in the English language with no time restrictions. Our search was conducted between November 2022 and March 2023. Both relative pre-clinical and clinical studies, as well as relevant reviews and meta-analyses were included. We additionally screened the references of the selected articles for possible additional articles in order to include most of the key recent evidence. We used the terms “Parkinson’s disease”, “depression”, “depressive”, “neuropsychiatric”, “mood”, “genetic”, “gene polymorphisms”, “genotype”, “allele”, “SNP”, “pathophysiology”, “pathway”, “mechanism”, and “mutation”, in different combinations. For narrative synthesis, our initial categorization was based on the various gene polymorphisms having been clinically investigated in PD depression, followed by a discussion on the potential pathophysiological mechanisms implicated in this relationship for each genetic factor, mainly based on in vitro and/or in vivo evidence.

## 2. Parkinson’s Disease Depression: Pathophysiological Aspects

Although the exact pathophysiological mechanisms underlying depression in PD remain elusive, it has been demonstrated that a combination of altered brain structure and connectivity, neurotransmitter imbalance, neuroinflammation, and dysregulation of neurotrophic factors contribute to its pathogenesis [22]. Main brain circuits suggested to be implicated in PD-related depression involve the orbitofrontal cortex–basal ganglia–thalamic and the basotemporal limbic circuit, in which the orbitofrontal cortex is connected with the temporal cortex via the uncinate fasciculus [22].

Impaired dopamine, 5-hydroxytryptamine (5-HT or serotonin), and noradrenaline neurotransmitter systems have been implicated in PD depression. Even though the most effective drugs against PD depression are antidepressants with both serotonergic and noradrenergic mechanisms of action, antiparkinsonian medications that are mainly developed for motor manifestations might also benefit depressive symptoms [22].

Serotonin is generated by serotoninergic neurons in the raphe nuclei in the brainstem, while serotoninergic projections reach almost the entire brain. Serotonin exerts a modulatory role and interacts with several neurotransmitter systems, thereby affecting numerous and diverse biological functions, such as mood, cognition, food intake, sleep, and motor activity. Serotoninergic neurotransmission has been involved in the pathophysiology of PD depression. Apart from the dopaminergic neurons in the SNpc, the serotoninergic neurons in the dorsal raphe nucleus also degenerate [23] and Lewy bodies are also present in these neurons in PD [24]. A postmortem study has revealed greater loss of serotoninergic neurons in the raphe nucleus in PD patients with depression compared to those without depression [25], suggesting a potential implication of serotonin neurotransmission in PD-related depression.

Given the relationship between dopamine and reward systems, as well as the impaired dopaminergic activity in PD in nigrostriatal pathways, but also in frontal and limbic areas, it has been hypothesized that impaired dopamine neurotransmission may be majorly implicated in PD-related depression [22]. In this context, an association between depressive symptoms in PD and dopaminergic neuronal loss in the ventral tegmental area has been proposed [22]. Reduced dopamine metabolism has been observed in the bilateral caudate and putamen, which was associated with the severity of PD depression [26], whereas there is also evidence depicting increased dopamine transporter (DAT) concentration in the striatal region in PD patients with depression compared to patients without depression [27].

The locus coeruleus, the primary noradrenergic brain region, also degenerates in PD; PD patients with depression display more pronounced neurodegeneration in this area compared to those without depression [28]. Furthermore, the levels of 3-methoxy-4-hydrophenylglycol, a noradrenaline metabolite, have been shown to be lower in the cerebrospinal fluid of PD patients independently of concurrent depression compared to non-PD patients with depression, indicating that noradrenergic neurotransmission may play a distinct role in PD-related depression [29].

Neurotransmitter transporters modulate the synaptic dopamine and serotonin levels and variations in genes encoding these transporters, but their receptors, other proteins, and enzymes implicated in their synthesis and metabolism have been proposed to affect the risk of depression in PD [30]. Furthermore, the role of polymorphisms in genes implicated in the regulation of neurotrophic factors and brain connectivity in PD depression has also been examined.

## 3. The Relationship between Genetic Forms of PD and Depression: A Brief Overview

Regarding the prevalence of depression among genetic forms of PD, excluding those with *GBA1* mutations, a recent systematic review has demonstrated that depression was more common in PD patients with *SNCA* triplication [6], while SNCA-Rep1 (CA)12/12 variant has been correlated with a lower risk for depression among PD patients [31]. Depression is particularly frequent in PD patients with *Parkin (PARK2)* mutations, while their compound heterozygotes’ non-PD relatives display an increased susceptibility to depression compared to the relatives not carrying this mutation [32]. The frequency of depression was shown not to significantly differ between *PINK1* mutation carriers and idiopathic PD [6]. Concerning *GBA* mutations, another systematic review has demonstrated that the rs76763715, rs387906315, rs421016, and rs80356773 variants were related to more depressive symptoms in PD [30]. Depression has been reported to be more frequent in *LRRK2* rs34637584 allele PD carriers compared to non-carriers, although there is also evidence that does not confirm this finding [30].

## 4. Genetic Factors Associated with Depression in Idiopathic Parkinson’s Disease: Insights into Molecular Mechanisms

**Figure 1:**
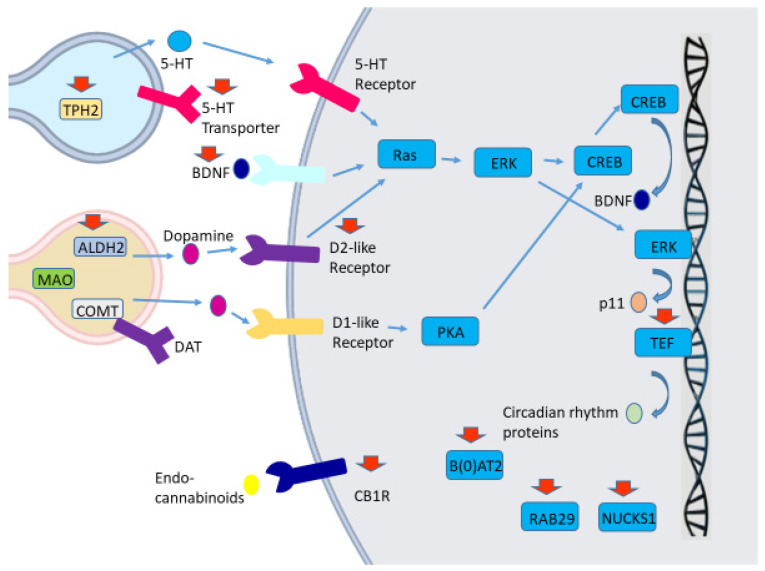
Molecular mechanisms potentially associated with the implication of genetic polymorphisms in Parkinson’s disease depression. Activation of serotoninergic, dopaminergic receptors results in several signaling pathways, which lead to the upregulation of a brain-derived neurotrophic factor (BDNF). Tryptophan hydrolase-2 gene (*TPH2*) is the main brain-specific rate-limiting enzyme involved in serotonin (5-HT) biosynthesis, while a serotonin transporter removes serotonin from the synaptic cleft, thereby majorly regulating synaptic serotonin levels and the degree of serotoninergic neurotransmission. Polymorphisms in *TPH2* and serotonin transporter genes have been associated with depression risk in PD. Monoamine oxidase (MAO) and catechol-O-methyl transferase (COMT) are the main enzymes that metabolize dopamine. In case of an MAO-mediated metabolism, 3,4-dihydroxyphenylacetaldehyde (DOPAL) is initially generated, which can be subsequently metabolized by aldehyde dehydrogenase (ALDH) to 3,4-dihydroxyphenylacetic acid (DOPAC). The *ALDH2* gene polymorphisms have been related to depression in PD. Dopamine receptors (DR) are divided into five subcategories (1–5), with DR1 and DR5 acting as D1-like receptors, and DR2, -3, and -4 acting as D2-like receptors. *Dopamine receptor D3 (DRD3)* gene polymorphisms may influence the risk of depression in PD patients. Endocannabinoids acting via cannabinoid receptors type 1 (CB1R) may also be implicated in PD depression pathophysiology, and polymorphisms in the gene encoding CB1R may affect PD depression risk. TEF is a circadian rhythm-related protein, and *TEF* gene polymorphisms have also been related to PD depression. BDNF upregulates the expression of p11, which is implicated in the transportation of serotonin receptors and other proteins to the cell membrane. BDNF plays a pivotal role in neuronal survival, growth, and differentiation. BDNF gene polymorphisms may also affect PD depression risk. The genes encoding the sodium-dependent neutral amino acid transporter B(0)AT2 and *PARK16* locus (RAB29 and NUCKS1) have also been associated with PD depression, although the molecular mechanisms remain unclear. Red arrows show the proteins whose encoding genes have been related to PD depression.

In the following section we discuss the genetic polymorphisms that have been associated with PD depression, along with their potential molecular mechanisms (Figure 1).

### 4.1. Genes Encoding Monoamine (Mainly Serotonin and Dopamine) Transporters

#### 4.1.1. Sodium-Dependent Serotonin Transporter Gene (*SLC6A4*) Polymorphism

Monoamine transporters consist of DAT, serotonin (SERT), and norepinephrine (NET) transporters, and they belong to the solute carrier 6 (SLC6) family [33]. This family includes proteins that transfer their substrates actively into the cells via the transmembrane electrochemical gradient. SLC6 proteins also transfer Na^+^ by symport, together with their substrates [33].

The sodium-dependent serotonin transporter, encoded by the *SLC6A4* gene on chromosome 17q11.1-17q12 [34], removes serotonin from the synaptic cleft, thereby majorly regulating synaptic serotonin levels and the degree of serotoninergic neurotransmission [35]. The 5-HT transporter constitutes a pharmacological target of several antidepressants, including selective serotonin reuptake inhibitors (SSRIs) and tricyclic antidepressants [36]. It has been demonstrated that a functional 44-base pair repeat insertion/deletion in the promoter of the *SLC6A4* gene (5-HTT-linked polymorphic region, known as 5-HTTLPR) can regulate 5-HT transporter expression levels. The most widely studied *SLC6A4* alleles are the 14-repeat, known as the long (L), and the 16-repeat, known as the short (S), alleles. In addition, there are other rarer variants, including the 15-, 19-, 20-, and 22-repeats [37]. The L allele enhances approximately threefold the expression of the gene compared to the S allele [36,38]. The 5-HTTLPR may influence the development of the brain cortex, as well as the function of circuits connecting the amygdala with the perigenual cingulate, thereby affecting depression risk [39].

In this regard, the presence of the S variant has been linked to depression and to a higher suicide risk after stressful events [40], seasonal affective disorder, atypical depression [41], and post-stroke depression [42]. In addition, the LS and SS 5-HTTLPR genotypes have been linked to anxious personality traits, especially in the elderly population [43]. However, no significant association has been observed between *SLC6A4* 5-HTTLPR variant and depression in a recent large study using samples from multiple populations [44].

Given the involvement of serotonin neurotransmission in PD depression, the role of *SLC6A4* gene polymorphisms has been investigated in the case of the PD-related depressive symptoms. In this context, it has been demonstrated that the S allele was significantly more frequent in PD patients with anxiety and depression compared to PD patients without these mood disorders [36]. In this small study, all PD patients suffered from the tremor-dominant PD phenotypic subtype, which raises concerns about the generalizability of these results. A larger study using a hospital-acquired sample and including patients with tremor-dominant and akinetic-rigid PD phenotypes has also shown that depressive symptoms were significantly more frequent in PD patients carrying two S alleles, compared to homozygotes for the L allele [45]. In agreement with the abovementioned evidence, a recent large study has also confirmed the protective role of the L homozygosity against depression in PD patients in the Chinese population [37].

On the contrary, no association has been identified between the S and L 5-HTTLPR polymorphisms and depression in PD in three other studies [14,46,47]. Methodological discrepancies, including the different sample sizes, the different ethnic groups examined, the hospital-acquired versus community-acquired sampling, the different scales used to detect depressive symptoms in PD patients, and the inclusion of patients receiving antidepressant treatment as mentioned in some of the abovementioned studies [46], might at least partially explain these inconsistent results. No significant associations were detected between 5-HTTLPR polymorphisms and PD depression in a meta-analysis [48]. However, a more recent meta-analysis has indicated that the S allele of the 5-HTTLPR polymorphism is associated with a higher risk of depression in PD [34], further supporting the role of this variant in the development of PD-related depressive symptoms. In the subgroup analysis of this study based on ethnicity, it was demonstrated that the S allele was associated with PD depression risk only for the non-Caucasian, and not Caucasian, populations in the recessive model. On the other hand, the S allele was not significantly associated with PD depression for both Caucasian and non-Caucasian subgroups in the dominant or additive model. Hence, it seems that the S allele might increase the risk for depression in PD mainly in the non-Caucasian populations.

Apart from the S and L alleles, the relationship between other known *SLC6A4* gene polymorphisms and PD depression has also been explored. In this regard, no associations have been identified between the variable number tandem repeats (VNTR) polymorphisms of the second intron and depressive symptoms in PD [47]. In addition, no significant relationships were found between several adjacent variations of single nucleotide polymorphisms (SNPs), named as tagging SNPs (tSNPs), of the *SLC6A4* gene and PD-related depression [47]. Furthermore, the rs25531 SNP of the *SLC6A4* gene, which may also affect gene expression, has not been associated with depression risk in PD in two studies [14,37]. Therefore, it seems that apart from the L/S alleles, other polymorphisms of the *SLC6A4* gene may not play a significant role in the onset of depression in PD. However, given the fact that the functional behavior of the G variant (rs25531) of the L allele resembles that of the lower activity S allele of the *SLC6A4* gene, it has been suggested that the impact of this SNP on the risk of PD depression should be also considered in order to avoid the masking of relative associations between L and S alleles and PD depression [14,49]. In this regard, a study that examined the influence of A/G SNP of the *SLC6A4* gene in relation to 5-HTTLPR and PD-related depression found no significant associations [14].

The potential role of serotonin and the serotonin transporter, particularly in PD depression, is not well understood. In a postmortem study, serotonin and its transporter have been indicated to be lower in the caudate nucleus of PD patients than in controls [50]. Higher serotonin transporter binding has been shown in limbic areas and raphe nuclei of PD patients with depression compared to those without depression, via positron emission tomography (PET) [51]. Furthermore, given the potential contribution of the S allele, which reduces the serotonin transporter gene expression in PD depression, it could be speculated that the possible upregulation of the serotonin transporter in these studies may reflect a compensatory mechanism. In agreement with this assumption, another study has indicated that serotonin transporters were not influenced in the midbrain of PD patients with depression, compared to those without depression at a relatively early stage of the disease [52]. Hence, disease staging is another important factor that should be considered in future studies in order to elucidate the potential underlying mechanisms.

Interestingly, it has been shown that chronic stress-induced depressive-like behavior in 1-methyl-4-phenyl-1,2,3,6-tetrahydropyridine (MPTP)-treated mice is associated with reduced expression of 5-HT receptor genes, further supporting the role of serotoninergic neurotransmission in the pathophysiology of PD depression [53]. However, how chronic stress may affect *SLC6A4* gene expression in PD depression remains to be explored.

The administration of venlafaxine, a serotonin-norepinephrine reuptake inhibitor (SNRI), has been recently demonstrated to reduce motor and depressive symptoms in rotenone-induced rat models of PD [54]. These behavioral improvements were accompanied by a reduction in the accumulation of α-synuclein, preservation of dopaminergic neurons, increased expression of the survival gene bcl-2 and the brain-derived neurotrophic factor (BDNF), regulation of apoptosis, autophagy, and the ubiquitin proteasome system, as well as the restoration of dopamine, serotonin, and noradrenaline levels. Hence, it can be proposed that genetic polymorphisms associated with serotoninergic neurotransmission in PD depression might influence several molecular mechanisms, including apoptosis, autophagy, neurotrophic factors, and α-synuclein accumulation, and this hypothesis remains to be further examined.

Collectively, genetic polymorphisms of serotonin transporters, and the L and S alleles, have been proposed to play a direct role in the development of depression in PD, with the S allele increasing the risk of PD depression, whereas the role of other polymorphisms of the *SLC6A4* gene remains more elusive.

#### 4.1.2. Dopamine Transporter Gene (*SLC6A3*) Polymorphisms

Dopamine reuptake in the striatum is mediated by the *SLC6A3* gene and its polymorphisms [55]. DAT determines to a great extent the dopamine levels in brain regions with high DAT density, including the striatum and the midbrain [33]. *SLC6A3* gene polymorphisms have been shown to affect PD risk in some studies [56], and a recent meta-analysis confirmed that 10-repeat allele may protect against PD development [57]. The levels of DAT in the striatal and limbic areas, reflecting anterior presynaptic dopaminergic impairment, have been demonstrated to be lower in depressed PD patients compared to non-depressed ones [58]. Polymorphisms in the DAT gene (*DAT1*/*SLC6A3*), present on chromosome 5p15.3, have been linked to visual hallucinations [59] and cortico-striatal activity during the execution of set-shifts in patients with PD [55]. *SLC6A3* genetic variations have been associated with depression risk in non-PD populations in some but not all studies [33]. The dopamine genetic risk score, developed by the combination of gene polymorphisms implicated in dopamine neurotransmission including DAT, has been associated with the risk of depressive symptomatology in non-PD populations [60]. In this context, the role of the SLC6A3 gene in PD depression has also been investigated.

A common 10-repeat variant of the 40-base pair VNTR polymorphism of the *SLC6A3* gene has been associated with a higher DAT activity compared to the 9-repeat variant [61]. Increased DAT activity causes lower synaptic dopamine levels and subsequently reduced dopamine availability [61]. Interestingly, it has been demonstrated that PD patients who were homozygous for the *SLC6A3* 10/10 repeat genotype displayed lower activity in the orbitofrontal cortex, which is related to reward outcome, in the Blood Oxygen Level Dependent (BOLD) functional magnetic resonance imaging (fMRI), compared to PD patients who were non-homozygous for the *SLC6A3* 10/10 genotype [62]. Since fronto-striatal activity related to reward may be linked with neuropsychiatric PD symptoms, including impulsivity, apathy, and depression [62], it could be proposed that the *SLC6A3* 10/10 genotype or other *SLC6A3* gene polymorphisms might be associated with PD-related depression. However, no significant associations have been demonstrated between several *SLC6A3* tSNPs and PD-related depression [47], indicating that *SLC6A3* may not play a significant role in PD depression risk; however, future studies are needed to confirm these data.

Importantly, it has been shown that the influence of the 10-repeat variant of the VNTR *SLC6A3* gene polymorphism on the risk of PD depends on sex. In particular, the homozygote 10-copy genotype of this gene has been associated with a reduced risk of PD only in males [63]. Therefore, sex might be considered as a possible modifier of PD depression risk as well.

### 4.2. Tryptophan Hydrolase-2 Gene (TPH2) Polymorphisms

Apart from the serotonin transporter, the role of other genes related to the serotoninergic pathway, such as tryptophan hydrolase-2 gene (*TPH2*), has also been investigated in PD depression. TPH2 is the main brain-specific rate-limiting enzyme participating in serotonin biosynthesis. A relative meta-analysis has shown that two *TPH2* SNPs, rs17110747 and rs4570625, are associated with major depressive disorder [64]. A 6-hydroxydopamine (6-OHDA) injection and the following levodopa treatment have been related to lower TPH2 levels in the midbrain of PD animal models [65]. *TPH2* polymorphisms have been linked with addictive behavior in PD [66]. However, in regard to PD depression, it has been demonstrated that acute tryptophan depletion could not influence the mood state of non-depressed PD patients [67].

Interestingly, the *TPH2* rs78162420 AC genotype has been correlated with a higher probability of depression in PD patients [68]. Although the molecular mechanisms remain to be explored, it has been shown that oxidative stress may inhibit TPH2 enzymatic activity, subsequent serotonin synthesis, and lead to the generation of TPH2 aggregates [69]. The redox status of the cysteine residues of TPH2 determine the catalytic activity of the enzyme, and reduced activity is related to the number of oxidized cysteines [69]. Since oxidative stress is greatly involved in PD pathogenesis, it could be hypothesized that *TPH2* gene polymorphisms may affect TPH2 aggregation and subsequently serotonin availability, thereby being implicated in PD depression. Moreover, in vivo evidence has shown a significant reduction in serotoninergic neurons in the dorsal raphe nucleus in Sim1-/- newborn mice, and TPH2 was demonstrated to be regulated by Sim1 [70]. Hence, Sim1-mediated TPH2 regulation might represent at least one of the underlying mechanisms of the effects of *TPH2* genetic variations in the depression related to PD.

### 4.3. Dopamine Receptor D3 Gene (DRD3) Polymorphisms

Dopamine receptors (DRs) are divided into five subcategories (1–5), with DR1 and DR5 acting as D1-like receptors, and DR2, -3, and -4 acting as D2-like receptors [71]. The *dopamine receptor D3 (DRD3)* gene, located on 3q13.31S chromosomal region, is primarily expressed in the globus pallidus (GP) and ventral striatum of the basal ganglia, where it mainly acts by modulating the release and clearance of dopamine [72]. Moreover, DR3 is implicated in emotional processes in the limbic system by receiving dopaminergic inputs originating from the ventral tegmental area [73].

Ser9Gly represents the most widely investigated *DRD3* gene polymorphism. The Gly-9 allele increases the affinity of DR3 to dopamine [74], enhances the dopamine-mediated cyclic adenosine 3′,5′-cyclic monophosphate (cAMP) response, and prolongs mitogen-associated protein kinase (MAPK) signaling, compared to the Ser-9 allele [75]. Carrying Gly alleles has been associated with major depression [76]. *DRD3* Ser9Gly polymorphism has been associated with neuropsychiatric conditions in PD in some studies, including impulse control disorder [74], behavioral addictions [77], and aberrant decision-making [78]. The higher affinity of DRD3 in the case of Gly carrying could subsequently disrupt reward-risk evaluation in the mesolimbic areas, thereby mediating impulsive behaviors [74].

Notably, a recent study has demonstrated that anhedonia and depressive symptoms were significantly more common in PD patients carrying Gly/Gly or Ser/Gly genotypes, compared to those with Ser/Ser genotypes of the Ser9Gly polymorphism in the *DRD3* gene [79]. In addition, Ser9Gly polymorphism of the *DRD3* gene could also affect the resting state (rs) brain function in patients with PD, being dependent on the severity of depressive symptoms [79]. In particular, the amplitude of low-frequency fluctuation (ALFF) values, which indicate the degree of regional functional neuronal activity in the rs-functional magnetic resonance imaging (MRI), in the right medial frontal gyrus were elevated in PD patients with Gly/Gly or Ser/Gly or genotypes when compared to those with Ser/Ser genotype [79]. ALFF values were also positively correlated with the severity of depressive symptoms and anhedonia in Gly carriers with PD [79]. The medial frontal gyrus belongs to the right medial prefrontal cortex, which is greatly involved in neuronal circuits implicated in emotional function and reward-risk evaluation [80]. Hence, *DRD3* Ser9Gly polymorphism may be correlated with the development of depression and anhedonia in PD, potentially reflected by the impaired neuronal activity in the medial frontal gyrus.

Regarding the potential underlying mechanisms, it has been demonstrated that D3R deficiency in the nucleus accumbens of mice is associated with depressive-like behavior, and restoration of D3R could improve depressive symptoms by possibly inhibiting microglia activation via the Akt signaling pathway [81]. In addition, lipopolysaccharide (LPS)-induced depressive-like behavior in mice has been shown to be accompanied by DR3 reduction in the nucleus accumbens, ventral tegmental area, and medial prefrontal cortex; pretreatment with pramipexole, acting as a preferential DR3 agonist, could exert antidepressant activity by preventing alterations in LPS-induced pro-inflammatory cytokines (tumor necrosis factor-α, TNF-α, interleukin (IL-)-1β, and IL-6), BDNF, and extracellular signal-regulated kinases 1/2 (ERK1/2)- cAMP response element-binding protein (CREB) signaling pathway [82]. Thus, neuroinflammatory pathways, BDNF regulation and the ERK1/2-CREB axis might represent underlying mechanisms affected by the diverse *DRD3* gene polymorphisms.

### 4.4. Monoamine Oxidase A (MAOA) and B (MAOB) Gene Polymorphisms

Monoamine oxidase (MAO) and catechol-O-methyl transferase (COMT) are the main enzymes that metabolize dopamine. In the case of an MAO-mediated metabolism, 3,4-dihydroxyphenylacetaldehyde (DOPAL) is initially generated, which can be subsequently metabolized by alcohol dehydrogenase/aldose reductase to form 3,4-dihydroxyphenylethanol (DOPET), or aldehyde dehydrogenase (ALDH) to 3,4-dihydroxyphenylacetic acid (DOPAC). DOPAL and DOPAC intermediate metabolites have been indicated to contribute to neurotoxicity; therefore, altered MAO activity might be implicated in the neurodegenerative process of PD.

A polymorphism in the 59-flanking regulatory region of the *MAOA* gene has been linked with affective disorders, including depression. In particular, it has been demonstrated that the short variants, 2 and 3, in the promoter region may increase the risk of depression and panic disorder, compared to the high activity long variants, 3a, 4, and 5 [83]. However, no significant associations have been detected between depressive symptoms and the *MAOA* gene’s short and long variants in patients with PD [45].

In addition to dopamine, MAO-B also degrades norepinephrine, which is implicated in several physiological functions, including cognition, pain, sleep, and emotional processing. Hence, MAO-B could mediate the pathophysiological mechanisms underlying the non-motor symptoms in PD, such as depression [8]. Recently, a study of PD patients from China and the Parkinson’s Progression Markers Initiative (PPMI) cohort has indicated that although the *MAOB* rs1799836 polymorphism was linked to the progression of non-motor symptoms of the patients in general in the Chinese cohort, there was no specific association with the progression of depression or anxiety in both cohorts [8]. Hence, the *MAOA* and *MAOB* gene polymorphisms seem not to significantly affect depression in PD patients.

### 4.5. Catechol-O-Methyltransferase Gene (COMT) Polymorphisms

Since the dopamine metabolites DOPAL and DOPAC, produced via MAO oxidation, may be neurotoxic, it has been proposed that decreased activity of COMT, which also metabolizes dopamine, may lead to elevated accumulation of these metabolites [84]. A *COMT* SNP, rs4680(A), has been related to significantly lower activity of the COMT enzyme [85]. It has been demonstrated that *COMT* gene polymorphisms may be associated with impaired executive function in PD patients [86], although there is also evidence not confirming the relationship between *COMT* polymorphisms and other non-motor symptoms in PD patients, such as impulse control disorder or daytime somnolence [87]. Although *COMT* SNP rs4680(A) has been correlated with worse motor function in PD patients, no significant differences were detected for depressive symptoms [84].

### 4.6. Aldehyde Dehydrogenase 2 Gene (ALDH2) Polymorphisms

Decreased activity of ALDH2 may lead to higher DOPAL levels, which have been shown to be neurotoxic [84]. An *ALDH2* SNP, rs671(A), specific to the Asian population, has been shown to reduce the enzymatic activity of *ALDH2* compared to the rs671(GG) SNP [88]. The co-occurrence of *ALDH2* rs671(GG) and *ADH1B* rs1229984(GG) alleles has been recently related to anxiety, depression, and alcohol-related disorders [89], and the *ALDH2* rs671(A), in combination with the *MAOA*-uVNTR (variable number of tandem repeat located upstream) 4-repeat variants, could reduce the risk of anxiety, depression, and alcohol dependence [90].

A previous study has demonstrated that PD patients with the GG genotype of *ALDH2* rs671 displayed increased scores in the “depressed mood” item of the Movement Disorder Society-Unified Parkinson’s Disease Rating Scale (MDS-UPDRS) [84]. The potential underlying mechanisms remain unknown. However, it has been demonstrated that the administration of Alda-1, an ALDH2 activator, may improve depressive symptoms in animal models of depression by lowering TNF-α and increasing mRNA levels of peroxisome proliferator-activated receptor-gamma coactivator, PGC-1α, a regulator of mitochondrial biogenesis in the hippocampus and frontal cortex of animals [91]. PGC-1a is majorly implicated in the modulation of mitochondrial biogenesis and emerging evidence suggests that it may participate in PD [92]. Hence, *ALDH2* gene polymorphisms may alter PD depression risk via mitochondrial and inflammatory pathways and future studies in this direction are needed.

### 4.7. Brain-Derived Neurotrophic Factor Gene (BDNF) Polymorphisms

BDNF has been involved in the neurobiology of both depression and PD [22]. BDNF is broadly present in the brain, and it plays a pivotal role in the survival of neurons, their growth, and differentiation. Experimental evidence suggests that it may protect against MPTP-induced neurotoxicity in vivo [93]. Reduced BDNF levels have been identified in the SNpc, as well as in the serum of PD patients compared to controls [94,95]. Interestingly, BDNF levels have been shown to be significantly reduced in the serum of PD patients with depression compared to those without depression [94], although a recent meta-analysis did not observe significant associations between serum BDNF levels and depressive symptoms in PD [95].

The Val66 replacement by Met66 (c.196G > A) of the *BDNF* gene, one of the most widely studied polymorphisms, disrupts the intracellular distribution of the protein, reduces its extracellular release, and subsequently its availability in the brain [96]. The Met allele can induce long-term depression (LTD) in the brain, thereby decreasing neuronal plasticity. This mechanism is pathologically implicated in depression, and the *BDNF* Met allele has been related to depression in older non-PD individuals [97]. The impact of the *BDNF* G196A polymorphism as a risk factor for PD onset remains unclear; however, it has been associated with cognitive decline and levodopa-induced dyskinesias in patients with PD [96,98].

No significant associations have been identified between *BDNF* G196A polymorphism and various motor and non-motor symptoms, including depression, in PD patients in two studies [96,99]. However, the Val allele of this gene polymorphism has been related to more severe anxiety and depression in PD patients in another study, as assessed by the self-reported Beck Anxiety Inventory (BAI) and Beck Depression Inventory (BDI) scales, respectively [100]. On the contrary, mild behavioral impairment (MBI) in PD has been shown to be significantly more frequent among *BDNF* Met allele carriers compared to Val homozygotes [101]. MBI is characterized by new and persistent neuropsychiatric symptoms in older individuals, and it has been correlated with a higher cognitive impairment risk. In particular, the abnormal thoughts/perception and emotional dysregulation subdomains of the MBI Checklist used to assess MBI were more severely impaired in PD patients carrying the Met allele in this study [101], suggesting that the *BDNF* Met allele may increase the risk of affective or psychotic symptoms in PD patients. These contradictory results might be explained by the different scales used for the assessment of neuropsychiatric symptoms, and the fact that the study by Cagni and colleagues included patients with an early-onset case and a positive family history.

The molecular mechanisms of BDNF contribution in the pathophysiology of PD depression remain largely unknown [22]. BDNF mediates its effects primarily via its binding to BDNF and Neurotrophin-3 (NT-3) receptors and the subsequent upregulation of several pathways, such as the Akt–glycogen synthase kinase-3 (GSK-3) and the MAPK-MAP kinase kinase (MEK) axes, which promote the gene expression of p11, a key molecule implicated in the transportation of serotonin receptors and other proteins to the cell membrane [22]. The expression of p11 is downregulated in depressed patients and in in vivo models of PD [22]. Furthermore, lithium, acting as a GSK-3 inhibitor and widely used for refractory depression and bipolar disorder, has also been shown to play a role in PD [22]. Therefore, MAPK- and GSK-3-related pathways may mediate the effects of BDNF activity in PD depression, and their role should be further investigated.

### 4.8. Cannabinoid Receptor Gene (CNR1) Polymorphisms

The endocannabinoid system (ECS) is involved in various physiological and pathophysiological mechanisms in the whole human body including the brain, where it is associated with mood, pain, and cognition among other functions [102]. ECS encompasses the G-protein-coupled cannabinoid receptors type 1 (CB1Rs) and 2 (CB2Rs), the endocannabinoids acting as CB1R and CB2R endogenous ligands, 2-arachidonoylglycerol (2-AG) and anandamide (AEA), and a group of molecules involved in their production, transportation, and degradation [102]. CB1R is encoded by *CNR1* gene in the 6q14–q15 chromosomal region [103]. CB1R is broadly expressed throughout the brain, predominantly in the basal ganglia, neocortex, substantia nigra, hippocampus, limbic structures, and cerebellum [104]. CB1R consists of 472 amino acids, and it belongs to the G-protein-coupled receptor superfamily coupled to inhibitory G proteins (Gαi/o), containing seven transmembrane hydrophobic domains [105]. CB1R activation by endocannabinoids affects neurotransmission in presynaptic nerve terminals, mainly by regulating gamma-aminobutyric acid (GABA)ergic and glutamatergic neurotransmitter pathways.

Several studies have shown that ECS plays an important role in the neuronal function and neurotransmission in the basal ganglia, including the striatum and GP [106]. The expression of CB1Rs has been demonstrated in the axon terminals of the glutamatergic cortico-striatal neurons, the glutamatergic neurons projecting to subthalamic nucleus (STN), globus pallidus internus (GPi)/substantia nigra pars reticulata (SNpr), and the GABAergic neurons innervating GPi/SNpr [106]. CB1R activation can alter GABA reuptake and the release of glutamate in the basal ganglia by modulating both the excitatory and inhibitory inputs into the SNpr and GPi [107]. In the striatal region, dopamine D1 and D2 receptors, as well as serotonin 5-HT1B receptors, are colocalized with CB1Rs, suggesting a potential structural or functional interaction between these neurotransmission systems [108].

Given their wide expression in brain regions that are highly affected in PD, as well as their critical implication in neurotransmitter imbalance, ECS and CB1R have been proposed to be involved in the neurobiology of PD. In this regard, CB1R’s levels have been demonstrated to be higher in the basal ganglia of humans in a post-mortem study, and in MPTP-treated marmosets compared to controls [109].

CB1R is involved in mood disorders, including depression. Increased CB1R mRNA levels have been identified in the limbic system of rats, implying its role in emotional regulation [110]. Drug abuse, including the use of cannabinoids, upregulates dopamine concentration in the mesolimbic regions of the brain, such as the nucleus accumbens [111]. D-9-tetrahydrocannabinol (THC), the active cannabis compound that interacts with C1BR in humans, has been indicated to reduce the release of serotonin, noradrenaline, GABA, and dopamine from neurons [112]. CB1R has been shown to be upregulated in the dorsolateral prefrontal cortex of suicide victims with major depression [113].

The (AAT)n polymorphism in the 3′ region of the *CNR1* gene has been considered as a regulator of gene expression, thereby exerting functional effects. Longer AAT repeat expansions can produce a Z-shaped DNA conformation, thereby potentially downregulating gene transcription [111]. In this context, the *CNR1* SNPs rs6454674 and rs806367 have been indicated to increase vulnerability to major depressive disorder and resistance to antidepressant treatment [114]. However, a recent meta-analysis did not reveal an association between *CNR1* rs1049353 or AAT repeat polymorphism with depression risk [115].

Concerning the implication of *CNR1* gene polymorphisms in the vulnerability to PD and PD-related depression, Barrero and colleagues (2005) have demonstrated that the length of the triplet (AAT)n of the *CNR1* gene was not significantly different between PD patients and controls, as well as between non-PD individuals with and without depression, although the sample size of the depressed non-PD participants was rather small [111]. On the other hand, the existence of at least one short *CNR1* gene allele carrying less than 16 AAT repeats was correlated with a higher frequency of depression among PD patients in this study, suggesting that ECS may be majorly implicated in the pathophysiology of PD depression [111]. However, since the length of AAT repeats may depend on the patient’s ethnic background [111], this association should be examined in other populations too.

The molecular mechanisms underlying the potential relationship between ECS and depression in PD remain to be further explored. CB1R can inhibit cAMP signaling pathways, and is, thus, involved in several cellular mechanisms and neuronal functions [106]. C1BR also plays a regulatory role in neuroinflammatory responses, excitotoxicity, oxidative damage, neurogenesis, and mitochondrial function, which represent crucial cellular mechanisms implicated in PD pathogenesis [106]. It is also possible that CB1R dysregulation may be indirectly implicated in PD-related pathogenesis, via its interaction with other neurotransmitters. Therefore, targeting of CB1R-mediated neurotransmission may represent a novel pharmacological approach to treating PD depression. Nevertheless, the neurobiological mechanisms of the potential role of CB1R and *CNR1* gene polymorphisms in PD-related depression remain to be explored.

### 4.9. TEF, CRY1, and CRY2 Gene Polymorphisms

*TEF*, *CRY1,* and *CRY2* genes are critically implicated in the regulation of circadian rhythms. In particular, the modulation of circadian rhythm is mediated by auto-regulatory feedback loops of the transcription and translation of circadian gene networks [116]. *BMAL1* and *CLOCK/NPAS2* genes constitute the positive feedback part, with their products brain and muscle ARNT-Like 1 (BMAL1) and CLOCK generating heterodimers that upregulate *PER1*, *PER2*, *CRY1*, and *CRY2* gene expression. During an approximate period of 24 h, PER and CRY proteins accumulate and produce heterodimers, which downregulate their own expression via their interaction with BMAL1/CLOCK [116]. Thyrotroph embryonic factor (TEF), is a transcription factor of the proline and acidic amino acid-rich basic leucine zipper (PAR bZip) family, which plays a major role in circadian rhythm regulation [117]. TEF expression can be enhanced by exposure to light, resulting in higher PER expression levels [116].

Impaired circadian rhythm has been linked to the pathophysiology of depression, clinically reflected by sleep disturbances, temperature changes, and altered rhythmic release of hormones [118]. The effectiveness of light therapy, sleep deprivation, and SSRIs used for depression treatment is at least partially mediated through the regulation of circadian rhythm [117,118]. *TEF*, *CRY1,* and *CRY2* gene polymorphisms have been associated with depression risk in non-PD individuals [119].

Concerning PD depression, it has been indicated that *TEF* rs738499, but not *CRY1* rs2287161 or *CRY2* rs10838524 gene polymorphisms, were linked to depressive symptoms among PD patients in the Chinese population [117]. More specifically, depression was significantly more common in PD patients carrying the TT genotype of *TEF* rs738499 [117], suggesting that this polymorphism may represent a genetic risk factor for PD-related depressive symptoms.

Although the neurobiological mechanisms underlying the connection between *TEF* gene polymorphisms and PD depression are not well understood, PAR bZip deficiency has been associated with lower serotonin and dopamine levels in the brains of mice [120], suggesting that TEF-mediated alterations in these transmitters might be implicated. Furthermore, the role of other circadian rhythm genes, including *BMAL1*, *CLOCK/NPAS2*, and *SIRT1*, in PD depression remains to be elucidated.

### 4.10. Sodium-Dependent Neutral Amino Acid Transporter B(0)AT2 Gene (SLC6A15) Polymorphisms

The *SLC6A15* gene, encoding sodium-dependent neutral amino acid transporter B(0)AT2, has been shown to affect susceptibility to major depression [121]. In patients with major depressive disorder, the *SLC6A15* rs1545843 A allele has also been associated with white matter integrity in the parahippocampal cingulum, a brain region involved in emotional regulation [122]. The *SLC6A15* rs1545843 AA genotype has been associated with depression in PD patients [68]. A recent study via proteomics methodology showed that SLC6A15 activity is related to mitochondrial function, neurite outgrowth, and oxidative stress [123], suggesting that these cellular functions may be involved in the effects of *SLC6A15* gene polymorphisms in PD depression.

### 4.11. PARK16 Genetic Locus Polymorphisms

The *PARK16* genetic locus on chromosome 1 contains five coding regions, and it was one of the first genetic factors linked to PD susceptibility by GWAS [124]. In particular, *PARK16* polymorphisms, such as RAB29 and NUCKS1, have been associated with a lower risk of PD in the Caucasian and Asian population [125]. A recent study has demonstrated that the progression of anxiety and depressive symptoms in PD patients was associated with the *PARK16* haplotype in a sample from the PPMI cohort [8], although the molecular mechanisms remain unclear.

In Table 1 we summarize the main findings of the clinical studies investigating the genetic polymorphisms that have been associated with PD depression.

## 5. Future Perspectives and Therapeutic Implications

In addition to the contribution of dopaminergic and serotoninergic neurotransmission in PD-related depression, acetylcholine may be involved. Depression has been linked to cholinergic denervation in the cortex in PD patients, regardless of their cognitive status [126]. In addition, lower α4β2-nicotinic acetylcholine receptor binding has been observed in the fronto-parieto-occipital cortex and cingulate cortex in PD patients with depressive symptoms [127]. The rs1044396 polymorphism in the *CHRNA4* gene, which encodes the neuronal nicotinic acetylcholine receptor α4 subunit, has been associated with depression in older individuals [128]. Hence, the role of this polymorphism in PD-related depression should be examined.

Neuroinflammation has also been associated not only with PD pathogenesis itself, but also with PD depression. For instance, high levels of the pro-inflammatory cytokine TNF-α have been observed in PD patients with depression and other non-motor symptoms, including cognitive impairment [129]. In another study, depressed PD patients displayed reduced cerebrospinal fluid interleukin (IL)-6 levels compared to non-PD depressed individuals [29]. Although no significant relationships have been detected between depression and *TNF-α* G-308A polymorphisms in a recent meta-analysis [130], their role in PD-depression deserves further study. Furthermore, the IL-1 type I receptor has been shown to affect the recovery of DAT levels and serotoninergic innervation in the striatum induced by MPTP in mice [131]. Given the potential impact of polymorphisms in genes related to IL-1 pathways on PD risk [132], a possible role between IL-1-related genes and monoamine neurotransmission could be proposed.

As mentioned above, *ALDH2* rs671 acts as a genetic modifier for non-PD-related depressive symptoms, especially in the presence of polymorphisms of other genes such as *MAOA* and *ADH1B* [89,90]. Therefore, another important aspect that should be taken into account is the potential interaction between specific gene polymorphisms in regard to PD-related depression risk.

The presence of depression has also been associated with akinetic-rigid phenotype in PD, compared to tremor dominant phenotype [17,111]. However, although akinesia-bradykinesia is majorly related to reduced dopamine levels, tremor has a less direct association with dopaminergic neurotransmission [111]. Interestingly, parkinsonian tremor may be linked to serotonin dysregulation, as it has been demonstrated in some functional neuroimaging and post-mortem studies [37,133]. In this context, it has been recently demonstrated that the AA genotype of the rs25531 variant of the *SLC6A4* gene could increase the risk of tremor in PD patients, compared to the GG or AG genotype [37]. Therefore, it would be useful to separately consider tremor-dominant and akinetic-rigid PD clinical phenotypes in future studies, in order to elucidate the impact of neurotransmitter systems on the relationship between relative genetic factors and depression in PD.

Regarding potential relationships between exposures and environmental factors, it has been demonstrated that although smoking may protect against the PD development [134], PD depression is positively associated with a smoking history [47]. In this regard, the *CB1R* rs2023239 variant has been shown to influence the reinforcing impact of nicotine in humans [135]. In addition, the use of non-aspirin based non-steroidal anti-inflammatory drugs (NSAIDs) has been linked to a higher frequency of depressive symptoms in PD patients [47], further supporting the potential role of inflammation-related gene polymorphisms in PD depression. S allele carriers of the *SLC6A4* gene have been shown to be more vulnerable to depression after stressful life events compared to non-carriers, indicating a possible gene-environment interaction [40]. In non-PD populations, *SLC6A3* SNPs can also interact with perinatal or environmental factors, including maternal rejection, to affect the risk of depression and suicidal ideation [33]. Furthermore, a meta-analysis has demonstrated that mutations in the *SLC6A3* gene were significantly higher in PD patients with exposure to pesticides compared to healthy controls, suggesting that pesticide-induced *SLC6A3* gene mutations may increase the risk of PD [136]. In this context, the impact of pesticide induced *SLC6A3* gene mutations on the development of PD depression could be further explored.

Epigenetic regulation of *SLC6A3* is crucially implicated in PD pathophysiology, although its exact role in the progression of non-motor symptoms remains largely unexplored. For instance, DNA methylation of the 5′-UTR region of the *SLC6A3* gene has been associated with PD development and progression [137]. The investigation of DNA methylation of other genes related to PD depression might also unravel epigenetic modifications as an important mechanism contributing to its pathogenesis.

Interestingly, depression has also been correlated with earlier age at onset of PD symptoms [47], although there is also evidence that does not confirm this hypothesis [138]. Given the potential diverse genetic background between early-onset and late-onset PD patients, stratification of PD patients according to onset age of the disease in future studies might unmask possible associations between genetic factors and depressive symptoms. In this regard, the *BDNF* G196A polymorphism has been linked with a later onset of PD [139], and especially geriatric depression in non-PD individuals [97].

Concerning other non-motor symptoms, depression has been associated with sleep disturbances, autonomic dysfunction, and cognitive impairment in PD [15]. Therefore, the contribution of gene polymorphisms known to be related to these non-motor manifestations could also be explored in the case of PD-related depression. For instance, the TT genotype of the TEF rs738499 is a shared independent genetic risk factor for both depression and sleep disturbances in PD [117,140].

Imaging genetics represent a novel approach that combines neuroimaging and genetic data in order to investigate the genetic contribution to clinical phenotypes, using neuroimaging features as an intermediate phenotype. This method is considered more sensitive than GWAS, since it aims to identify the impact of genetic factors and, subsequently, the potential underlying neurobiological mechanisms of the development of clinical phenotypes, for example, depression, through the additional integration of regional neuroimaging patterns. In this regard, a recent study using imaging genetics has identified eighteen SNPs, including *LOC284395*, *RAB15*, and *NRXN1* genes, that may be correlated with the severity of depressive symptoms in PD patients [141]. In detail, neuroimaging features were obtained via tractography of diffusion tensor imaging, and SNPs were selected based on their known association with non-PD-related depression. In this study, depressive symptoms were associated with structural connectivity in the anterior cingulate cortex, hippocampus, and amygdala. Interestingly, the *LOC284395* gene has been related to the function of amygdala and anterior cingulate cortex, while *RAB15* is known to be implicated in hippocampal function [141]. It would be of particular interest for future studies using imaging genetics approaches to investigate the impact of SNPs that have been associated with PD or PD-related depression on depressive symptoms in PD, since they may aid in the elucidation of the underlying pathological mechanisms of PD-related depression.

Moreover, dopaminergic or antidepressant treatment may affect the neurotransmitter tone in relative brain regions of PD patients, and a potential interaction between treatment effects and genotype should also be considered. In regard to serotoninergic neurotransmission, it has been demonstrated that, in advanced PD, levodopa can be converted to dopamine by serotoninergic neurons that project to the striatal region, resulting in a possible displacement of serotonin in these serotoninergic nerve terminals and impaired serotonin metabolism [22].

Concerning potential therapeutic implications, the *SLC6A4* S allele has been related to a worse response to SSRIs in geriatric patients with major depression [142]. In addition, the interaction between *TPH2* methylation status and ALFF of the left inferior frontal gyrus in the fMRI has been shown to affect the short-term antidepressant treatment response in patients with major depressive disorder [143]. The *SLC6A3* variants have also been associated with a higher effectiveness of levodopa for motor symptoms [144], although their impact on the efficacy of antidepressant treatment for PD depression remains unclear. Furthermore, pramipexole, a dopaminergic agonist widely used for PD motor symptoms, exhibits anti-depressive effects via DRD3 in animal models of PD [145]. It has also been demonstrated that genetic variations in the *SLC6A3*, *SLC6A4*, and noradrenaline transporter genes affect the atremorine-mediated dopamine responses in PD [146]. Atremorine is a potent dopamine enhancer that may act neuroprotectively in PD. Hence, gene polymorphisms in monoamine transporters might influence the effectiveness of dopaminergic therapy as well, with potential impact on PD-related depression.

Furthermore, ethnic differences in allele distributions should be taken into consideration. For instance, the frequency of the SS 5-HTTLPR allele is lower in Caucasians compared to Asians; in the Caucasian population, the S allele frequency is lower than that of the L allele, whereas in the Han Chinese population, the S allele is more common than the L allele [34].

The cross-sectional design of most studies described above constitutes an important limitation, since several PD patients might have developed depression at a later stage of the disease. Hence, future prospective longitudinal studies with larger sample sizes and age-matched controls taking into account disease duration would be of particular value. In addition, the selection of appropriate scales assessing depressive symptoms and optimal cut-off points; the stratification of patients, whenever possible, into early-onset and late-onset PD, as well as tremor-dominant and akinetic-rigid PD phenotypes; the exclusion of patients with genetic forms of PD; the consideration of antidepressant medications; the detailed evaluation of other non-motor manifestations; and the consideration of different ethnic backgrounds would aid our deeper understanding of the pathophysiological mechanisms underlying the impact of genetic factors in PD-related depression.

## 6. Conclusions

In conclusion, emerging evidence highlights that several genetic polymorphisms implicated in serotoninergic and dopaminergic neurotransmission and metabolism and neurotrophic factors, especially BDNF, circadian rhythm, and the endocannabinoid system, may affect depression risk in PD (Table 1). Although the exact mechanisms underlying the potential role of genetic diversity in PD depression remain unclear, they may involve neurotransmitter imbalance, mitochondrial impairment, oxidative stress, neuroinflammation, and the dysregulation of neurotrophic factors and their downstream signaling pathways. However, given the partially contradictory results of the abovementioned studies, further evidence is needed in order to elucidate the exact molecular mechanisms underlying the impact of genetic factors in the pathophysiology of PD-related depression.

## Figures and Tables

**Table 1:** Clinical studies investigating the role of gene polymorphisms in Parkinson’s disease-associated depression.

Study	Gene	Evidence of Association with PD Depression	Reference
Menza et al., 1999	*SLC6A4*	The presence of S variant in 5-HTTLPR is correlated with depression and anxiety in PD patients.	[36]
Mossner et al., 2001		SS 5-HTTLPR genotype is correlated with depression in PD patients, compared to LL 5-HTTLPR genotype.	[45]
Wang et al., 2019		LL 5-HTTLPR genotype may protect against depression in PD patients. No significant associations observed between the rs25531 SNP and depression in PD patients.	[37]
Zhang et al., 2009		No significant associations observed between the S and L 5-HTTLPR polymorphisms, the rs25531 SNP, and their co-occurrence and depression in PD patients.	[14]
Burn et al., 2006		No significant associations observed between the S and L 5-HTTLPR polymorphisms and depression in PD patients.	[46]
Dissanayaka et al., 2011		No significant associations observed between the S and L 5-HTTLPR polymorphisms, the VNTR polymorphism, and several tSNPs and depression in PD patients.	[47]
Zheng et al., 2017	*TPH2*	The rs78162420 AC genotype is associated with depression in PD patients.	[68]
Dissanayaka et al., 2011	*SLC6A3*	No significant associations observed between several *SLC6A3* tSNPs and depression in PD patients.	[47]
Zhi et al., 2019	*DRD3*	Gly/Gly or Ser/Gly genotypes are associated with depression and anhedonia in PD patients, compared to Ser/Ser genotypes of the Ser9Gly polymorphism.	[79]
Mossner et al., 2001	*MAOA*	No significant associations observed between depression and the short and long variants in the 59-flanking regulatory region of the gene in PD patients.	[45]
Cui et al., 2022	*MAOB*	The *MAOB* rs1799836 polymorphism is associated with the progression of non-motor symptoms, but no specific association with the progression of depression or anxiety in PD patients.	[8]
Yu et al., 2021	*COMT*	No significant associations observed between SNP rs4680(A) and depression in PD patients.	[84]
Yu et al., 2021	*ALDH2*	The GG rs671 genotype is associated with depression in PD patients.	[84]
Svetel et al., 2013	*BDNF*	No significant associations observed between G196A polymorphism and depression in PD patients.	[96]
Gao et al., 2010		No significant associations observed between G196A polymorphism and depression in PD patients.	[99]
Cagni et al., 2017		The Val allele of G196A polymorphism is associated with more severe anxiety and depression in PD patients.	[100]
Ramezani et al., 2020		The Met G196A allele is associated with mild behavioral impairment and, in particular, impaired emotional dysregulation and abnormal thoughts/perception subdomains compared to Val homozygotes among PD patients.	[101]
Barrero et al., 2005	*CNR1*	The presence of at least one short *CNR1* gene allele with less than 16 AAT repeats is associated with depression in PD patients.	[111]
Hua et al., 2012	*TEF*	The TT rs738499 polymorphism is associated with depression in PD patients.	[117]
Hua et al., 2012	*CRY1*	No significant associations observed between *CRY1* rs2287161 and depression in PD patients.	[117]
Hua et al., 2012	*CRY2*	No significant associations observed between *CRY2* rs10838524 and depression in PD patients.	[117]
Zheng et al., 2017	*SLC6A15*	The rs1545843 AA genotype is associated with depression in PD patients.	[68]
Cui et al., 2022	*PARK16* genetic locus	The *PARK16* haplotype is associated with the progression of depression and anxiety in PD patients.	[8]

## Data Availability

Not applicable.
